# PDEGEM: Modeling non-uniform read distribution in RNA-Seq data

**DOI:** 10.1186/1755-8794-8-S2-S14

**Published:** 2015-05-29

**Authors:** Yuchao Xia, Fugui Wang, Minping Qian, Zhaohui Qin, Minghua Deng

**Affiliations:** 1 Center for Quantitative Biology Peking University, Beijing 100871, China; 2 School of Mathematical Sciences Peking University, Beijing 100871, China; 3 Department of Biostatistics and Bioinformatics Emory University, Atlanta GA 30322, USA

## Abstract

**Background:**

RNA-Seq is a powerful new technology to comprehensively analyze the transcriptome of any given cells. An important task in RNA-Seq data analysis is quantifying the expression levels of all transcripts. Although many methods have been introduced and much progress has been made, a satisfactory solution remains be elusive.

**Results:**

In this article, we borrow the idea from the Positional Dependent Nearest Neighborhood (PDNN) model, originally developed for analyzing microarray data, to model the non-uniformity of read distribution in RNA-seq data. We propose a robust nonlinear regression model named PDEGEM, a Positional Dependent Energy Guided Expression Model to estimate the abundance of transcripts. Using real data, we find that the PDEGEM fits the data better than mseq in all three real datasets we tested. We also find that the expression measure obtained using PDEGEM showed higher correlation with that obtained from alterative assays for quantifying gene and isoform expressions.

**Conclusions:**

Based on these results, we believe that our PDEGEM can improve the accuracy in modeling and estimating the transcript abundance and isoform expression in RNA-Seq data. Additionally, although the stacking energy and positional weight of the PDEGEM are relatively related to sequencing platforms and species, they share some common trends, which indicates that the PDEGEM could partly reflect the mechanism of DNA binding between the template strain and the new synthesized read.

The PDEGEM model can be freely downloaded at: http://www.math.pku.edu.cn/teachers/dengmh/PDEGEM.

## Introduction

The transcriptome is the set of all RNA molecules in a cell including mRNA, rRNA, tRNA, microRNA and other non-coding RNA. Quantifying the expression level of mRNAs in a given cell is a fundamental problem in transcriptome research. Microarray was one of the most popular technologies to quantitatively measure gene expression in the past decade [1-4]. Despite its wide range of applications and successes, there are some key limitations in the microarray technology, for instance, poor estimation on low-expressed genes because of the effects of the cross-hybridization and background signal [5-8]. In addition, designing the array relies on the known sequence and annotation information, therefore novel transcripts are not able to be discovered and measured. An ultra-high-throughput sequencing-based technology, known as RNA-Seq [9], showed that it could overcome these difficulties [4], [7], [10-12]. Traditionally, for RNA-Seq data, reads are mapped to the reference genome or transcriptome or de novo assembled together to produce a genomescale transcriptional profile [13-15]. The profile can be summarized by a sequence of "counts", which stand for the number of reads whose mapping starts at that position [7]. A basic question is how to use these counts to quantify the gene expression for each transcript. From our experience in analyzing microarray data, we believe the key to more accurate and effective expression quantification is to establish an appropriate statistical model. Mortazavi et al. proposed to use Reads Per Kilobase per Million mapped reads (RPKM) to represent the expression level of the transcript [16]. The RPKM method gives full consideration to the transcript length and the number of mapped reads and has been widely used due to its simplicity. This method, however, ignores the variability of read coverage within each transcript hence introduced inaccuracy [13]. Since then, more sophisticated models have been proposed for expression quantification in RNA-Seq. Marioni et al. proposed to use a constant rate Poisson model [9], Risso et al. proposed a method to correct GC-content bias [17] and Vardhanabhuti et al applied a Bayesian model [18]. Li et al proposed a variable rates Poisson model, termed mseq, to fit the read count data. They applied two algorithms, Poisson-Linear model and MART, to implement the variable rates Poisson model. The mseq method takes into account the read count bias due to the sequence preference, therefore, it could fit the real data better.

Despite the improvement in mseq, it is oversimplified to just consider the neighborhood nucleotide sequence information alone. They also discussed the linear effect of dinucleotides in the Supplementary Material, which showed little improvement than the single-nucleotide model. However, we believe that a better approach is to consider the binding interaction between two adjacent nucleotides more precisely. An existing model that considers interaction between adjacent nucleotides is the Positional Dependent Nearest Neighborhood (PDNN) model [19], [20]. The PDNN model was originally designed to model the probe-probe interaction that frequently observed in the microarray data and the results are quite favorable. The fundamental idea of the PDNN model lies that the binding affinity of a probe can be approximated as a weighted sum of its stacking energy [3], [21], [22]. Inspired by the success of PDNN, we believed that the sequence contents may play an important role in affecting the base-level read counts in RNA-Seq experiments. Then we developed the PDEGEM, a Positional Dependent Energy Guided Expression Model to take into account the sequencing preference. The sequencing preference may lie in two aspects. The first one is the nucleotides before the starting position of reads, which could either affect the break point in random broken, or influence the amplification efficiency. The second part is the nucleotides after the starting points of reads, which may affect the binding affinity between the template strain and the generated strain. In this study, we mainly focused on the transcript abundance and isoform expression estimation, thus we only considered single-end reads, and the model could be easily extended to handle paired-end reads.

## Methods

### Positional Dependent Energy Guided Expression Model

We use a nonlinear PDNN model to characterize the non-uniformity of the read distribution due to systematic biological properties.

Let *n_ij _*represent the count of reads that start at the *j*th nucleotide of transcript *i*. In this model, we assume that the counts from isoforms are modeled as Poisson distribution, and counts in different position have different Poisson rates. That is *n_ij _~ P oisson*(*μ_ij _*), where *μ_ij _*is the rate of the Poisson distribution. This is the same definition as in mseq. For a given position of a transcript, we use the nucleotide sequences nearby to evaluate the sequencing preference of this position. We attempt to approximate its sequencing preference by the binding affinity estimated from applying PDNN on the nucleotide sequences nearby, which represents how strong a single-stranded DNA sequence can bind to its template sequence. According to PDNN, binding affinity is a function of free energy. And the free energy of a single-stranded DNA sequence *E_ij _*is characterized as the sum of weighted pair-wised stacking energies, which is defined in Equation 1.

(1) $$E_{ij}=\overset{N-1}{\underset{k=1}{\sum }}w_{k}\in_{b_{k}},b_{k+1}$$

where *w_k _*is the positional weight factor depending on the position of the nucleotide in the sequence. The term *b*_*k *_, *b*_*k*+1 _is the neighborhood nucleotides that represent A, C, G, and T. $E_{b_{k},\;b_{k+1}}$ is the stacking energy indicating the interaction of two adjacent nucleotides, which is the same as the stacking energy used in the PDNN model. N is the number of nucleotides nearby that were considered (N/2 nucleotides upstream and N/2-1 nucleotides downstream, also including the given nucleotide itself) to evaluate the sequencing preference.

Next, according to PDNN model, the binding affinity of a single-stranded DNA sequence with N nucleotides *E_ba _*is defined in Equation 2.

(2) $$E_{ba}=\frac{1}{1+exp\left(\overset{N-1}{\underset{k=1}{\sum }}w_{k}\in_{b_{k},b_{k+1}}\right)}$$

*E_ba _*indicates the sequencing preference that affects the read count of a given nucleotide. Next, we combine the idea of PDNN and mseq and apply a nonlinear regression model to characterize the sequence preference, as shown in Equation 3.

(3) $$log\left(\mu_{ij}\right)=v_{i}+\alpha +log(\frac{1}{1+exp\left(\overset{N-1}{\underset{k=1}{\sum }}w_{k}\in_{b_{k},b_{k+1}})\right)}$$

where *ν_i _*= *log*(*μ_i_*), *μ_i _*is the expression of the *i*th transcript and *α *is a constant. *μ_ij _*is the rate of the Poisson distribution and *μ_ij _*= *w_ij _μ_i_*, where *w_ij _*is the sequencing preference, which may depend on the surrounding sequence and the stacking energy between two adjacent nucleotides. In this model, by default, N is chosen to be 40 (Details see Additional files). As a result, our model uses 39 positional weight parameters and 16 stacking energy parameters and 1 constant totally 39 + 16 + 1 = 56 parameters, which is highly dependent on N, to model the sequencing preference of a given nucleotide.

After that, *R*^2 ^is used to measure the fitness of the non-linear regression model. In PDEGEM, we define *R*^2 ^the same as mseq did, which is shown in Equation 4:

(4) $$R^{2}=1-\frac{d}{d_{0}}$$

where *d *is the deviance of the fitted model, and *d*_0 _is the deviance of the null model. The null model of PDEGEM is when we assume equal positional weight and use the initial stacking energy provided by PDNN model, i.e., $w_{k}=\frac{1}{N-1},k=1,2,\ldots ,N-1$. We apply cross validation to estimate *R*^2 ^by dividing the transcripts into five parts, with the first four parts to estimate the parameters, while using the left one part to calculate the *R*^2^. The higher *R*^2 ^is, the better the model could fit the data, thus the more reasonable the model is.

### Fitting PDEGEM

To fit PDEGEM, we optimize the following objective function (shown in Equation 5) using Newton Method with a penalty function to obtain the positive positional weight and stacking energy.

$$\text{min}\underset{i}{\sum }\underset{j}{\sum }{\left(log\left(\mu_{ij}\right)-v_{i}-\alpha -log\left(\frac{1}{1+exp\left(\overset{N-1}{\underset{k=1}{\sum }}w_{k}\in_{b_{k},b_{k+1}}\right)}\right)\right)}^{2}$$

In order to optimize the constrained nonlinear least squares, we add the logarithmic penalty function(shown in Equation 6), where *λ → *0 is the penalty parameter.

(6) $$\text{min}\underset{i}{\sum }\underset{j}{\sum }{\left(log\left(\mu_{ij}\right)-v_{i}-\alpha -log\left(\frac{1}{1+exp\left(\overset{N-1}{\underset{k=1}{\sum }}w_{k}\in_{b_{k},b_{k+1}}\right)}\right)\right)}^{2}$$

We perform a optimization strategy in order to get the optimal positional weight and the stacking energy

Step 1. $v_{i}=log\left(\overset{L_{i}}{\underset{j=1}{\sum }}\frac{n_{ij}}{L_{i}}\right)$ is initialized, where *L_i _*is the length of the transcript.

Step 2. For each position on a transcript, initialize the stacking energy $\in_{b_{k,}b_{k+1}},k=1,\ldots ,N-1,\in_{b_{k},b_{k+1}}=\frac{1}{N-1}$, to get an stacking energy vector, where N is the number of nucleotides surrounding the position. Then we merge the stacking energy vector of all positions on all transcripts in the training set to get a stacking energy matrix. Initialize positional weight $w_{k}=\frac{1}{N-1}$ to make each position the same initialized weight. Then we use Newton Method with the penalty function to obtain the optimal positional weight *w *= (*w*_1_, *w*_2_,..., *w*_*N *−1_). Next, utilize the optimal vector of positional weight *w *and take the stacking energy provided by PDNN as the initial value. Then we use Newton Method to iterate stacking energy ∈ = (∈_1_, ∈_2_,..., ∈_16_) and obtain an optimal vector of stacking energy *E*, which represents the energy weight of dinucleotides *AA, AC, ..., T T *.

Step 3. Compute and update *v_i _*= $v_{i}=log\left(\overset{L_{i}}{\underset{j=1}{\sum }}\frac{n_{ij}}{W_{i}}\right)$,

$$\text{Where}\;W_{i}=\frac{\overset{L_{i}}{\underset{j=1}{\sum }}n_{ij}}{\overset{L_{i}}{\underset{j=1}{\sum }}exp\left(\alpha +log\left(\frac{1}{1+exp\left(\overset{N-1}{\underset{k=1}{\sum }}w_{k}\in_{b_{k},b_{k+1}}\right)}\right)\right)}$$

Step 4. Utilize the optimal vector of positional weight and stacking energy in step 2 as the initial value of step 2. Alternate implementation of step 2 to step 3 until both positional weight and stacking energy minimize the objective equation. In the article, we use a threshold (0.1) and stop the iteration if the distance between adjacent two iterations is less than the threshold.

Step 5. Utilize the optimized positional weight and stacking energy to estimate the maximum likelihood estimation of the transcript abundance, which can be presented as in Equation 7.

(7) $$\mu =\frac{\overset{L}{\underset{j=1}{\sum }}n_{j}}{\overset{L_{i}}{\underset{j=1}{\sum }}exp(\alpha +log(\frac{1}{1+exp\left(\overset{N-1}{\underset{k=1}{\sum }}w_{k}\in_{b_{k},b_{k+1}})\right)})}$$

Where *L *is the length of the transcript, and *n_j _*(*j *= 1, 2,..., *L*) is the read count of the *j*th position.

## Results

### Datasets

In this study, four different RNA-Seq datasets (See Table 1) are used to compare the performance of PDEGEM with other methods, including RPKM [16] and mseq.

**Table 1:** Illustration of 3 datasets

Dataset	Subdataset		Platform	read length	mapping
Dataset 1	Wold	w1:Brain w2:Liver w3:Muscle	Illumina/Solexa	25 bp	Seqmap
	
	Burge	b1:Group 1 b2:Group 2 b3:Group 3	Illumina/Solexa	32 bp	Seqmap
	
	Grimmond	g1:EB g2:ES	SOLiD	35 bp	SOC
	
Dataset 2	Synthetic spike-in RNA-Seq		Illumina/Solexa	36 bp	Bowtie

Dataset 3	Two samples from human kidney Two samples from human liver		Illumina	36 bp	Bowtie

Dataset 4	Three samples from mouse liver Three samples from mouse Muscle Two samples from human Brain		SOLiD	25 bp	Seqmap

#### Dataset 1

This dataset consists of three sub datasets, named Wold data [16], Burge data [23], and Gimmond data [24] for short, which are originally utilized to validate the performance of the mseq method. We use these datasets to compare the performance of PDEGEM with the Poisson-Linear model and MART method implemented in mseq. The first two sub datasets are sequenced by Illumina's Solexa platform, while the third dataset is generated with ABI's SOLiD platform. In Wold data, the length of reads is 25 bp and the reads came from three mouse tissues: brain (w1), liver (w2) and skeletal muscle (w3). The Burge data consists of human tissues, mammary epithelial and breast cancer cell lines. They are divided into three groups. Group 1 (b1) is adipose, brain and breast. Group 2 (b2) is made of colon, heart and liver. Lymph node, skeletal muscle and testes are in group 3 (b3). The length of the read in Burge data is 32 bp. The Grimmond data is generated from two cell lines: embryonic stem cells (EB, g1) and undifferentiated mouse embryonic stem cells (ES, g2). The length of read in Grimmond data is 35 bp. In order to acquire a high qualitative dataset, some reads are truncated into 30 bp or 25 bp.

#### Dataset 2

This dataset is a synthetic spike-in RNA-Seq dataset created by the External RNA Control Consortium (ERCC) [25], which is designed to develop a set of RNA standards and includes 96 spike-in transcripts. The reads are generated using Illumina GAII, with 36 bp in length. We chose this dataset (library 6) because the transcripts had known initial concentration and they were also detected in the 100% ERCC RNA-Seq experiment. Thus taking the initial concentration as the golden standard, we can apply Spearman's rank correlation coefficients to compare the concordance between the initial concentration of transcripts and their abundance estimated using various RNA-Seq transcript abundance estimation methods.

#### Dataset 3

This dataset is originated from the Marioni et al. study [9], which consists of two samples from human kidney and two samples from liver tissues. We choose this dataset because these samples are profiled by both RNA-Seq and Affymetrix Microarray. The read length of Illumina RNA-Seq samples is 36 bp. We use the transcript abundance measured by microarray as the gold standard and compared with the transcript abundance obtained from RNA-Seq data estimated by different transcript abundance estimation methods.

#### Dataset 4

This dataset is originated from the Mortazavi et al. study [16], which consists of three mouse tissues: liver, skeletal muscle and brain are sequenced on the Solexa platform. We choose this dataset because these samples are profiled by both RNA-Seq and Affymetrix Microarray [26]. The read length of Solexa RNA-Seq samples is 25 bp. We used the isoform expression measured by microarray as the gold standard and compare with the isoform expression estimation obtained from RNA-Seq data estimated by different methods.

### Extracting the count data from the original reads

To evaluate the goodness-of-fit of mseq and PDEGEM, we apply the same strategy with mseq to compare *R*^2 ^of these two models. For each of the 8 sub-dataset in Data Set 1, we utilize the 100 highest expressed single-isoform transcripts selected by mseq as the training set to train the parameters.

For Dataset 2, we first map the reads of Spikein dataset to the reference using bowtie version 0.12.5 with the -best option and allow at most two mismatches (-v 2 option) [27]. For this dataset, we first remove all positions with zero read count and we truncate the 20 bp at either beginning or end of transcript.

For the RNA-Seq data in Dataset 3, we also apply bowtie version 0.12.5 with the -best option and allow at most two mismatches (-v 2 option) to map reads to Refseq transcripts (UCSC hg19) and collect the read count of each position of each transcript. Also, the 100 highest expressed single-isoform transcripts are chosen as the training set.

For the RNA-Seq data in Date Set 4, we apply SeqMap [28] with two mismatches and map all sequencing reads to the mouse reference sequences (mm9, NCBI, Build 37) and download gene annotations from the RefSeq mouse mRNA database(mm9, NCBI, Build 37)

### Goodness-of-fit of PDEGEM and mseq in Dataset 1

We apply PDEGEM to three sub-datasets in Data Set 1 and compared their *R*^2 ^with that of mseq. In Data Set 1, for each of the eight RNA-Seq samples, we select the 100 highest expressed single-isoform transcripts provided by mseq and use 5-fold cross-validation to train PDEGEM. That is, 75 isoforms are selected as train datasets randomly and the other which acquire the *R*^2 ^value are used as test datasets. In table 2 we compared the *R*^2 ^of the Poisson-Linear model and MART model with PDEGEM for each sub-dataset. Compared with the other two methods, we can see in the last column that PDEGEM achieves the highest *R*^2 ^in all eight samples, which indicates that PDEGEM can fit the RNA-Seq data much better than mseq. We also compare our model with Cufflinks and Genominator. The results see the supplementary.

**Table 2:** *R*^2 ^for 3 models in 8 RNA-Seq samples in Dataset 1.

*R* ^2^						
**Dataset**	**Sample**	**PL^1^**	**PL^∗2^**	**MART^∗2^**	**MLE**	**PDEGEM^2^**

Wold	Brain	0.51	0.65	0.70	0.68	**0.73**
	Liver	0.50	0.64	0.70	0.66	**0.70**
	Muscle	0.46	0.56	0.59	0.60	**0.71**

Burge	Group 1	0.42	0.49	0.52	0.53	**0.61**
	Group 2	0.35	0.42	0.46	0.50	**0.58**
	Group 3	0.42	0.50	0.54	0.52	**0.59**

Grimmond	EB	0.40	0.54	0.58	0.58	**0.60**
	ES	0.37	0.54	0.54	0.56	**0.58**

*R*^2 ^for 3 models in 8 RNA-Seq samples in Dataset 1. Eight different sub-datasets are chosen to compute the *R*^2 ^of these three models. We chose 40 nucleotides, 20 bp upstream, and 19 bp downstream to estimate the sequencing preference. For each row, the number in bold indicates the highest *R*^2 ^among different methods in for the dataset.

^1^No Cross-Validation.

^2^Cross-Validation.

^∗ ^PL: the Poisson-Linear model;

^∗ ^EB: Embryonic stem cells;

^∗ ^ES: Undifferentiated mouse embryonic stem cells.

### Trends of the stacking energy and positional weight in Dataset 1

**Figure 1:**
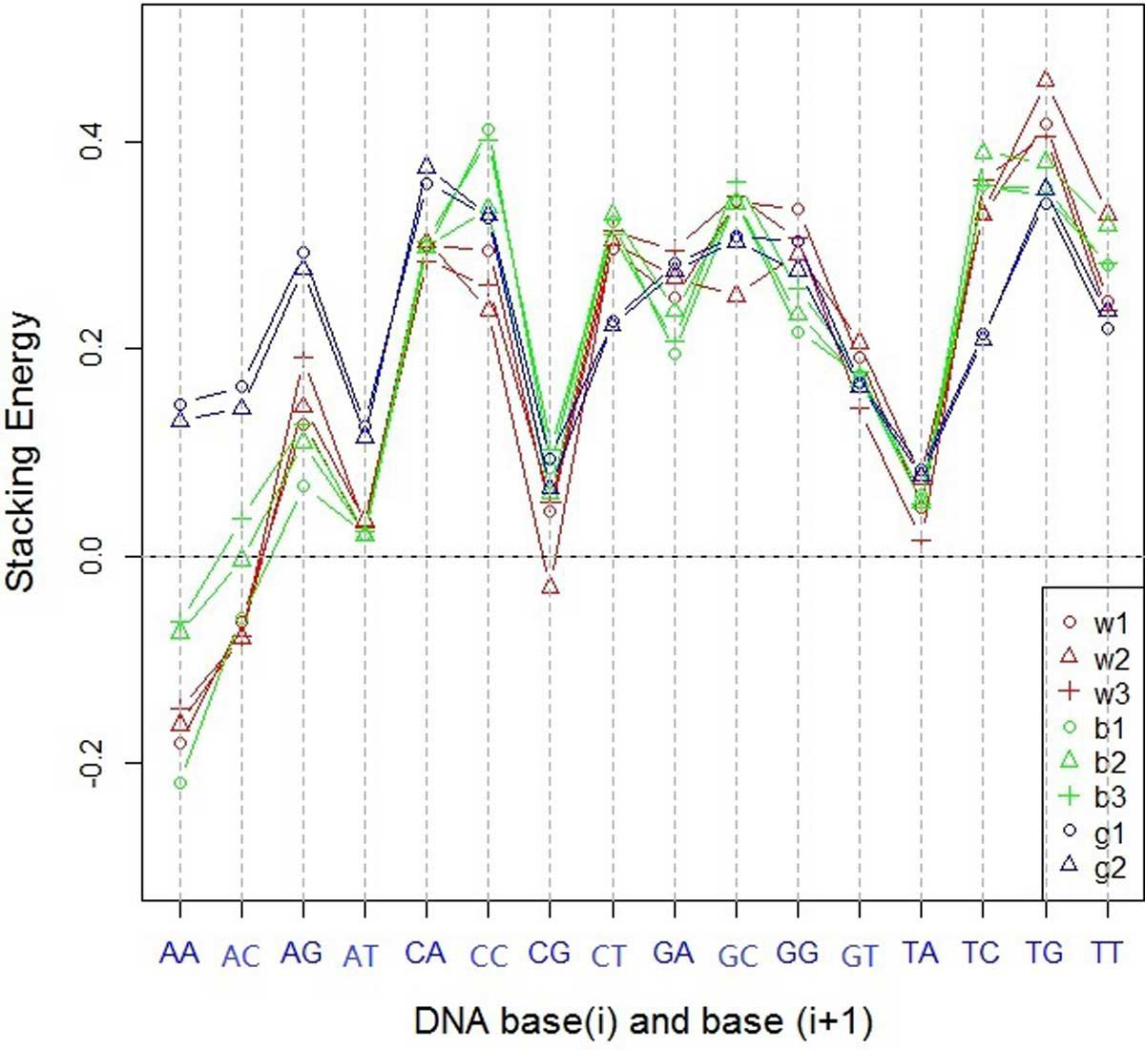
**The stacking energy of PDEGEM in 8 different samples of Dataset 1**. The x-axis represents the 16 dinucleotides *AA, AC, ..., and TT *, while the y-axis indicates the stacking energies of the dinucleotides. Lines with different colors indicate different datasets. w1, w2 and w3 represent Wold data, b1, b2 and b3 stand for Burge Data, while g1 and g2 indicate Grimmond data.

Figure 1 shows the stacking energy of PDEGEM in three sub-datasets in Dataset 1. From this figure, we can see that the stacking energies of the Illumina platform for different species shared similar trend (w1, w2, w3 for mouse and b1, b2, b3 for human in Figure 1), except for dinucleotides AA and AC, while it is slightly different for the SOLiD platform (g1 and g2 in Figure 1). The slight difference between stacking energy of mouse samples Wold data and Grimmond data might be caused by the different sequencing technologies. Although Illumia Soxlea and ABI SOLiD both apply sequencing by synthesis strategy to generated RNA-Seq reads, Soxlea platform could identify one nucleotide at each fluorescence scanning, while SOLiD could identify two nucleotides.

**Figure 2:**
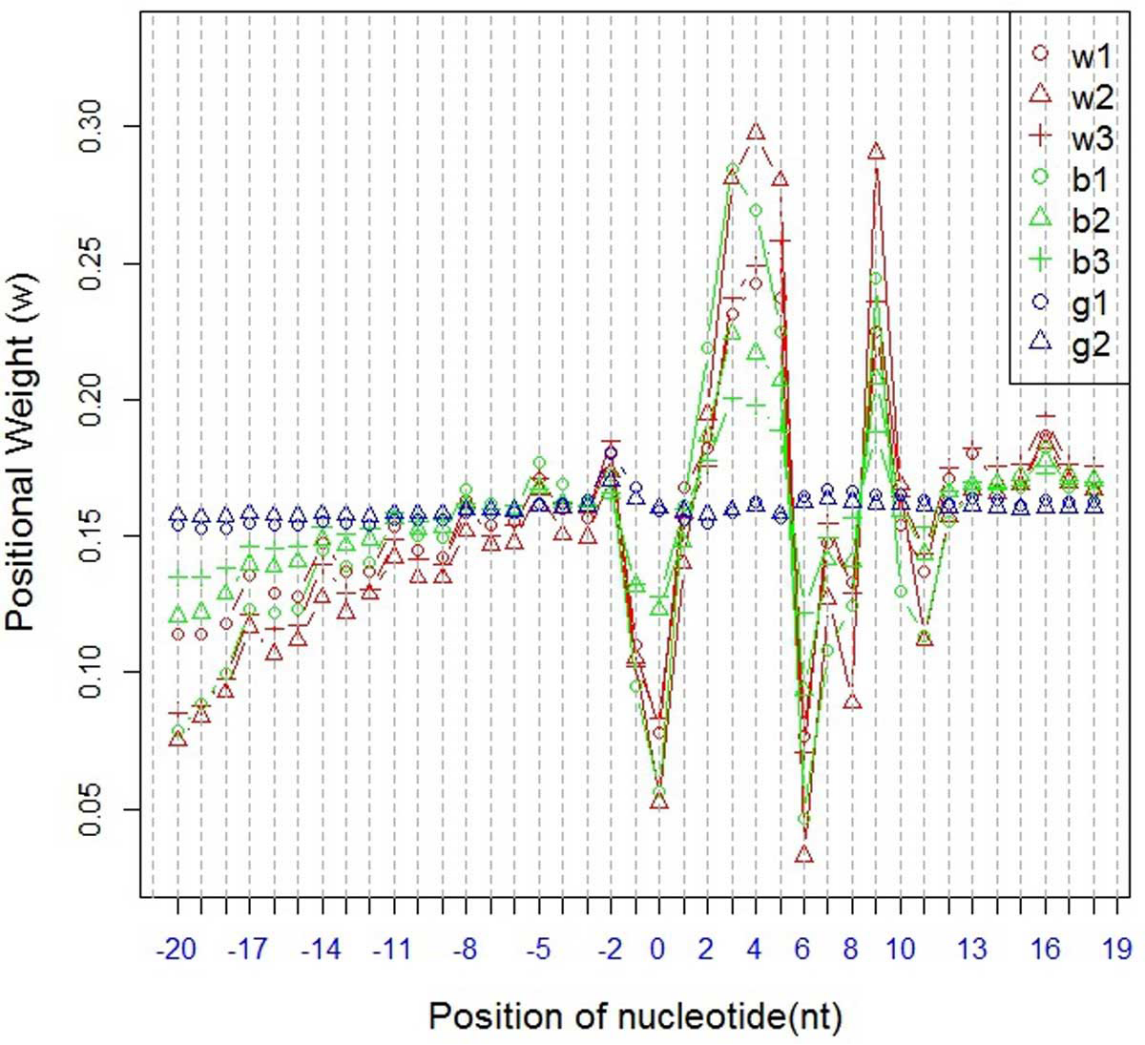
**The positional weight of PDEGEM in 8 different samples of Dataset 1**. We chose 40 surrounding nucleotides (20 upstream and 19 downstream) to fit the model. The x-axis is the relative position around the starting point of a read. The y-axis indicates the positional weight. Red lines (w1, w2, w3) represent Wold data, green lines (b1, b2, and b3) for Burge Data and blue lines (g1 and g2) for Grimmond data.

Figure 2 shows the positional weight of the three sub-datasets when using PDEGEM. From this figure, we could see that the nucleotides in the middle portion of the 40 bp sequence showed larger weights, which indicates the nucleotides around the starting point of the read have larger effect on the sequencing preference. In addition, we found that the positional weights of Wold data and Burge data that were generated from the Illumina platform shared the same trend (w1, w2, w3 and b1, b2, b3 in Figure 2), which is significantly different from that of Grimmond Data generated from the SOLiD platform (g1 and g2 in Figure 2). Based on the two figures, we could see that although the positional weight and stacking energy of PDEGEM are slightly related to the sequencing platforms and species, they share some common trends and are relatively conservative across species and sequencing platforms. We believe that PDEGEM could partly reflect the binding mechanism between two nucleotide sequences.

**Figure 3:**
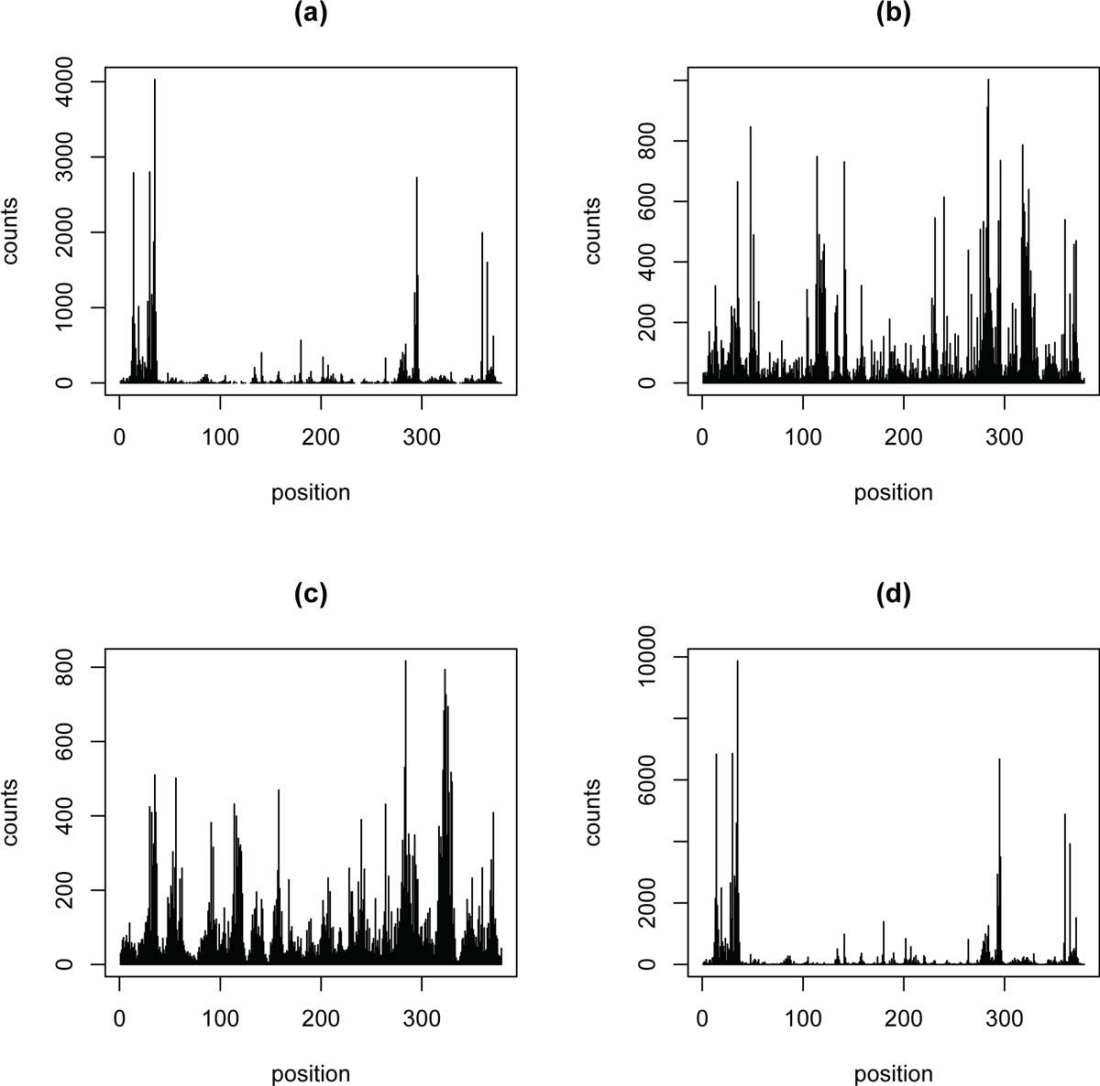
**Fitting counts for the mouse Rp19 gene**. Black vertical lines represent counts (experimental values or fitted values) along the Grimmond EB Rp19 gene (with the UTR and a further 100 nucleotides truncated). We use the other 99 genes of the top 100 genes to train the three models and then predict the counts for the Rp19 gene.(a) Counts of reads (true values) in the Grimmond EB data.(b) Counts of fitted reads using mseq. (c) Counts of fitted reads using MART. (d) Counts of fitted reads using PDEGEM.

Figure 3 shows an example of the prediction of the different methods perform. The four pictures of Figure 3 are the counts on gene Rp19 in the Grimmond EB of dataset1, counts fitted by the original data, PDEGEM, Poisson linear model and MART model respectively. The four pictures in Figure 3 indicate that PDEGEM can fit RNA-Seq data much better than other two methods.

### Comparison in synthetic RNA-Seq data

Utilizing the synthetic spike in RNA-Seq data in Data Set 2, we further evaluate the performance of RPKM, MART, and PDEGEM. We compared the Spearman's rank correlation coefficients between the transcripts abundance estimated by these models from the RNA-Seq data and the "true" transcript abundance indicated by the experimental transcript concentration. Presumably, the higher the correlation is, the more accurate the method is in estimating the transcript abundance from RNA-Seq reads. There are 96 transcripts in the spike-in dataset, of which six do not have reads mapped to. Thus the 90 transcripts left were used in the following analysis. We again chose the 40 highest transcripts measured experimentally as the training set to train the positional weight and stacking energy, and these parameters are used to measure the gene expression of the other 50 transcripts.

For the training dataset, cross-validation is used to compare the goodness-of-fit for MART and PDEGEM. We found that PDEGEM had higher *R*^2 ^(*R*^2 ^= 0.65) than MART (*R*^2 ^= 0.30). Next, we compare the Spearman's rank correlation coefficient of RPKM, MART, and PDEGEM with the true transcript abundance measured experimentally. Table 3 shows that the Spearman's rank correlation coefficient by PDEGEM is a little higher than the other two methods, which again indicates that PDEGEM could give a better fit of the RNA-Seq data than the two methods in mseq.

**Table 3:** Consistency between transcript abundance estimated by different methods and gold standards in Dataset 2.

	RPKM	MART	PDEGEM
*R*^2^	*−*^1^	0.30	**0.54**
SRCC^2^	0.8341	0.8501	**0.8655**

Spearman's rank correlation coefficient of RPKM, MART, PDEGEM with the true transcript abundance measured by transcript concentration in the experiment [25]. We compared four different methods using a synthetic RNA-Seq dataset with 90 isoforms. The number in bold indicates the highest *R*^2 ^among three methods for the dataset.

^1^RPKM has no *R*^2^.

^2 ^Spearman's rank correlation coefficient.

### Comparison in the Marioni dataset

First, four methods (RPKM, Poisson-Linear, MART, and PDEGEM) were chosen to estimate the transcript abundance of the four RNA-Seq datasets in Dataset 3. After that, we used the transcript abundance measured by the Affymetrix GeneChip as the gold standard to assess the performance of these four methods.

For the last three models, 40 nucleotides in the neighborhood of each nucleotide were used and the 100 highest expressed single-isoform genes were used as the training set. As shown in the last column of Table 4 PDEGEM also achieved the highest goodness-of-fit measured by *R*^2^.

**Table 4:** *R*^2 ^for 3 models in Dataset 3.

Sample	Poisson-Linear	MART	PDEGEM
SRX000571^1^	0.15	0.50	**0.61**
SRX000604^1^	0.12	0.48	**0.59**
SRX000605^2^	0.15	0.53	**0.60**
SRX000606^2^	0.13	0.52	**0.59**

Goodness-of-fit measured by *R*^2 ^for Poisson-Linear model, MART and PDEGEM in four RNA-Seq samples in Data Set 3. The four samples came from human kidney and liver. For each row, the number in bold indicates the highest *R*^2 ^among different methods for the dataset

^1^Illumina sequencing of Human liver transcripts.

^2^Illumina sequencing of Human kidney transcripts.

As highly expressed transcripts often display high level of over-dispersion as well as high spatial dependence [13], for each sample, we chose the 8000 highest expressed transcripts according to the microarray data. We only looked at the transcripts appear in both RNA-Seq and microarray data, after that we got rid of the transcripts whose RPKM are less than 0.1, as these transcripts have too few reads to detect the accurate abundance. Finally, about 5000 transcripts were left to compare the performance of these four methods. When compared to the gene expression measured by microarray, PDEGEM again achieved the highest Spearman's rank correlation coefficient in all four RNA-Seq samples is shown in Table 5. According to the results above, we have reasons to believe that PDEGEM could provide more accurate quantification of gene expression at transcript-level when compared with the other three methods.

**Table 5:** Consistency between transcript abundance estimated by different methods and gold standards in Dataset 3.

Sample	N^1^	RPKM	PL^2^	MART	PDEGEM
SRX000571^3^	4857	0.474	0.474	0.471	**0.483**
SRX000604^3^	4880	0.460	0.458	0.460	**0.477**
SRX000605^4^	5309	0.527	0.527	0.530	**0.557**
SRX000606^4^	5293	0.442	0.411	0.452	**0.471**

Spearman's rank correlation coefficient of RPKM, Poisson-Linear model, MART, PDEGEM with the "true" gene expression measured by microarray in Dataset 3.

^1 ^Number of transcripts used to calculate the correlation coefficients.

^2 ^PL: the Poisson-Linear model.

^3 ^Illumina sequencing of Human liver transcripts.

^4 ^Illumina sequencing of Human kidney transcripts.

### Comparison in the Mortazavi dataset

To compare the performance of PDEGEM with regard to exon-level estimations, three methods (uniform model, MART, PDEGEM) are chosen to estimate the isoform-specific expressions with those data profiled by Affymetrix Microarray given by Pan et al that studied 3126 'cassette-type' alternative splicing(AS) events in 10 mouse tissues. We use a percent alternatively spliced exon exclusion value (%Asex) as a statisitc presented by Jiang et al [29]. We use Dataset 4 which is the same subsets of genes in the paper by Jiang et al and utilize the method for estimating isoform expression levels introduced by Jiang et al to compute %ASex. The Pearson's correlation coefficients (PCC) in all subset of genes is shown in Table 6. According to the result in Table 6 PDEGEM achieved the highest PCC and we have reasons to believe that our model can improve the isoform-specific expression levels.

**Table 6:** Pearson's correlation coefficients of %ASex.

	Tissue	AS events	PCC^1 ^by uniform model	PCC by MART	PDEGEM
1	Brain	699	0.36	0.40	**0.41**
	Liver	472	0.48	0.50	**0.53**
	Muscle	451	0.40	0.45	**0.47**

2	Brain	298	0.44	0.50	**0.52**
	Liver	228	0.60	0.60	**0.62**
	Muscle	194	0.48	0.51	**0.52**

The Pearson's correlation coefficients(PCC) of uniform model, MART, PDEGEM with the isoform expression measured by microarray in Dataset 4.

^1^Pearson's correlation coeficient

## Discussion and Conclusion

Sequencing preference has been recognized as an important factor in transcript abundance estimation. We believe that the stacking energy between neighborhood nucleotides can be utilized to better model sequencing preference than in the Poisson-Linear model. Therefore, we borrow the idea from the PDNN model that is first introduced to model the binding affinity between the target sequence and probe on the microarray. Combining the ideas of these two models, we construct PDEGEM for analyzing the RNA-Seq data. We assume that the starting position of a read is related to two main effects. The first one is the effect of RNA amplification, including random broken and insufficient amplification, which is presented by the nearby nucleotides prior to the starting point of the reads. The second one is the related sequencing procedure, which is presented by the nucleotides after the starting point of the reads. As for Illumina Solexa platform, it utilizes sequencing by synthesis procedure to produce RNA-Seq reads. The affinity between the template strain and the generated strain may play an important role in generating the reads.

As for sequencing technology and species, we found that different platforms or species generate slightly different positional weight and stacking energy in our model. Similar difference has been noticed in microarray data. For stacking energy, except for AA, AC, CC, and GC, the other dinucleotides show the similar trend in both Illumina and SOLiD platforms(Figure 1). The stacking energies are slightly different between the Wold Data and Burge Data that generated from the same sequencing platform Illumina. This may due to the fact that the RNA samples were extracted from different species (mouse and human), which shows that both sequencing platforms and species could influence the sequencing preference through stacking energy. As for positional weight, the results show that the nucleotides in the middle portion of the sequence have larger effects on the sequencing preference. Besides, the Wold data and Burge data generated with Illumina show almost the same trend of positional weight (Red and green lines in Figure 2), regardless of the species. However, it's significantly different for Gimmond data that are generated from the SOLiD sequencing platform (Blue lines in Figure 2). Our results shows that the sequencing platforms may have larger effect on positional weight than species. The same trend is shown in Dataset 2 and Dataset 3 (Details see Additional file 2). Although the parameters in the model are slightly related to both sequencing platforms and species, they share some common trends, which are relatively conservative across sequencing platforms and species. Thus, it nevertheless reflects that the DNA binding mechanism can be identified between the template strain and the new synthesized read. In this study, we only observe that sequencing preference are slightly related to sequencing platforms and species, while the detailed mechanism remains to be further explored with more refined biological experiments.

Furthermore, we also attempt to use 20, 60, 80, and 120 nucleotides to characterize the sequencing preference (See Additional file 1 for more details). Take the RNA-Seq data w1 in Wold Data of Dataset 1 for example, the *R*^2 ^are almost the same as that of using 40 nucleotides. Besides, the stacking energy and positional weight are also very similar, as shown in Supplementary Figure S1. Therefore in order to reduce memory consumption, we chose to use 40 nucleotides in the PDEGEM. The detailed comparison is shown in the Supplementary Figure S1. In addition, the positional weight and stacking energy calculated from the spike-in RNA-Seq data generated from Illumina in Dataset 2 and four RNA-Seq samples generated with Illumina in Dataset 3 showed that they also share the similar trend as those of Wold data and Burge data in Data Set 1, which confirm the biological significance of the positional weight and stacking energy. More details could be found in Supplementary Figure S2 and Supplementary Figure S3.

In this study, we first calculate *R*^2 ^through cross-validation and the *R*^2 ^of PDEGEM increases significantly compared with Poisson-Linear model and MART. Also, PDEGEM is developed to estimate the gene expression at transcript level. By comparing with RPKM, Poisson-Linear model and MART, our method shows higher goodness-of-fit measured by *R*^2 ^with less parameters and achieves higher Spearman's rank correlation coefficients with gene expression measured by microarray or other experiments. We believe that the improvement in *R*^2 ^and transcript abundance estimation are the result of the more refined sequencing preference model.

In this model, we use Newton Method to solve the optimization equation. The convergence of Newton Method is time-consuming and this method may appear singular for particularly large amount of data. An alternative method that could be considered is the coordinate descent method. Though our model is based on single-end RNA-Seq protocol, paired-end RNA-Seq data can also be analyzed with PDEGEM.

PDEGEM characterize the non-uniformity of read distribution in RNA-Seq data. Through applying the model to RNA-Seq data generated with Illumina and SOLiD platforms, the results show that PDEGEM is relatively conservative to the sequencing platforms and species. PDEGEM combines two ideas, one is Poisson linear model which considers the sequencing preference, and the other is the PDNN model which considers the binding affinity to predict gene expression in microarray. The experimental evaluation of PDEGEM has illustrated that it can achieve higher goodness-of-fit and more accurate prediction of transcript abundance compared with other methods, which will surely benefit the downstream investigations such as detecting differentially expressed genes and gene set enrichment analysis.

## Competing interests

The authors declare that they have no competing interests.

## Authors' contributions

FGW and YCX designed the studies and developed statistical model. YCX wrote python and R program and performed data analysis and modeling. FGW downloaded datasets form web and processed datasets with bowtie. YCX and FGW wrote the manuscript. All authors review and revise the manuscripts.
